# Parents’ experiences with pediatric chronic pain

**DOI:** 10.1080/24740527.2019.1577679

**Published:** 2019-02-22

**Authors:** Anne Le, Bruce R Dick, Jude Spiers, Kathy Reid, Shannon D. Scott

**Affiliations:** a Faculty of Nursing, University of Alberta, Edmonton, Alberta, Canada; b Department of Anesthesiology and Pain Medicine, University of Alberta, Edmonton, Alberta, Canada; c Stollery Children’s Hospital, Alberta Health Services, Edmonton, Alberta, Canada

**Keywords:** pediatric chronic pain, parents, narrative, qualitative, chronic pain, pediatrics

## Abstract

**Introduction**: Pediatric chronic pain affects 15%–39% of children. Chronic pain can have significant negative effects on a child’s physical functioning, psychological and cognitive functioning, quality of life, and social functioning. Parents of children with chronic pain have reported being affected by their child’s condition. There have been few studies exploring the experiences of parents of children with chronic pain through a qualitative descriptive lens.

**Methods**: Thirteen parents from a pediatric chronic pain clinic participated in semistructured interviews. Concurrent data collection and analysis occurred to allow for follow-up of ideas that emerged during analysis. Three phases of analysis occurred: coding, categorizing, and developing themes.

**Results**: Three themes were developed: (1) Parents’ emotional journey; (2) chronic pain affects the entire family; and (3) social support is critical. Parents described emotions caused by the arduous process of obtaining a chronic pain diagnosis, followed by difficulties finding strategies to help their child manage the pain. Family life was affected because special accommodations often had to be made. Families were affected financially, incurring costs due to time off of work or additional therapies. Finally, parents stressed the importance of a strong social support network to provide assistance and flexibility for the changing needs of their child.

**Discussion**: This research identified a better understanding of the impact of pediatric chronic pain on parents. These findings can be used to provide and promote more effective treatments and education to improve the psychological, physical, and social well-being of children with pediatric chronic pain and their families.

## RÉSUMÉ

**Introduction**: La douleur chronique pédiatrique affecte de 15 % à 39 % des enfants. La douleur chronique peut avoir des effets négatifs importants sur le fonctionnement physique, psychologique et cognitif des enfants, de même que sur leur qualité de vie et leur fonctionnement social. Il a été établi que les parents des enfants souffrant de douleur chronique sont affectés par l’état de leur enfants. Il y a peu d’études portant sur le vécu des enfants souffrant de douleur chronique dans une optique descriptive analytique.

**Méthodes**: Treize parents d’une clinique de la douleur chronique pédiatrique ont participé à des entrevues semi-structurées. La collecte et l’analyse des données ont été effectuées de manière pour permettre de donner suite aux idées ayant émergé pendant l’analyse. Trois phases d’analyse ont été effectuées : la codification, la catégorisation et le développement des thèmes.

**Résultats**: Trois thèmes ont été développés : 1) le parcours émotionnel des parents; 2) la douleur chronique affecte la famille toute entière; et 3) le soutien social est primordial. Les parents ont décrit les émotions occasionées par le processus ardu d’obtention d’un diagnostic de douleur chronique, auquel s’ajoutent les difficultés pour trouver des stratégies permettant d’aider leurs enfants à prendre en charge leur douleur. La vie familiale était affectée étant donné que des mesures spéciales devaient souvent être prises.. Les familles étaient touchées financièrement et devaient assumer cetains coûts en raison de la perte de temps de travail ou des frais liés à des traitements aditionnels. Finalement, les parents ont fait état de l’importance d’un solide réseau de soutien social pour les aider et leur leur donner une certaine souplesse pour faire face aux besoins changeants de leur enfant.

**Discussion**: Cette étude a permis de mieux comprendre les répercussions de la douleur chronique pédiatrique sur les parents. Ces résultats peuvent être utilisés pour offrir et promouvoir une meilleure éducation ainsi que des traitements plus efficaces pour améliorer le bien-être psychologique, physique et social des enfants souffrant de douleur chronique pédiatrique et de leurs parents.

## Background

Chronic pain, defined as prolonged or persistent pain lasting longer than 3–6 months, is a significant clinical problem in pediatric populations.^1–4^ The origins of the pain can vary and may be caused by injury, chronic disease (e.g., arthritis), or be a chronic condition itself (e.g., fibromyalgia).^1^ Past research has demonstrated that pediatric chronic pain negatively impacts not only a child’s physical health but also many other aspects of his or her life, including psychological and cognitive functioning, school attendance and participation, quality of life, and social functioning.^2,3^ Furthermore, chronic pain has been linked to increased distress and depression in children and adolescents.^5^

Likewise, research has demonstrated that parents of children with chronic pain also experience negative emotional, mental, and social outcomes.^6–10^ Several studies assessing parents’ experiences have revealed increased emotional distress, anxiety, and frustration in coping and adapting to their child’s condition.^7,11,12^ In a study conducted by Hunfeld et al. to determine the impact of chronic pain on the quality of life of adolescents and their families, researchers found predictive values of pain for social functioning and mastery (coping strategy used by parent to help deal with the stresses of having a child with pediatric chronic pain).^12^ That is, mothers with children with more severe chronic pain described feeling more restricted in what they could do in their social lives and had more problems coping with their children’s pain compared to mothers with children with less severe chronic pain.^12^ The impact of pediatric chronic pain on parents suggests a need to understand parents’ experiences in caring for a child with pediatric chronic pain in order to develop knowledge translation tools that help parents cope with and navigate the onset, investigations, diagnosis, and treatment of pediatric chronic pain.

A few studies have centered on studying the experiences of parents in caring for a child with pediatric chronic pain.^6–9^ Noel and colleagues used cluster analysis to examine the pain narratives of parents of children with chronic pain in a therapeutic context. They characterized vulnerability-based and resilience-based aspects of pain narrative and found two styles of pain narratives, distress and resilience.^6^ Researchers found that parents who were categorized as having *distress n*arratives had higher anxiety and anger.^6^ Researchers limited the duration of narratives to a fixed period of time (first 15 min after narrative began), which may potentially limit the rich experiences of study participants. As a result, a study that utilizes methods that allow participants to share freely and as much as possible in order to obtain a full understanding of their experiences is needed.

Jordan et al. conducted focus groups with 17 parents and caregivers of adolescents with chronic pain and analyzed their data using interpretative phenomenological analysis (IPA).^13^ The researchers found two primarily negative themes that encompassed the impact of parenting a child with chronic pain: “struggle for control and coherence” and “a very different life, p. 51.”^7^ In a second study, Jordan et al. conducted semistructured interviews with fathers of adolescents with chronic pain and also analyzed the data using IPA. They identified four themes that were salient across all interviews: helplessness (due to not being able to alleviate their child’s pain), containment (ways of dealing with the challenges associated with the pain or “containing” the problem), balance, and re-evaluation (of their role in their child’s care).^8^ Their results mirror those of Noel et al. and suggest the need for more supports for parents as they help their child navigate the journey of pediatric chronic pain. Although IPA and cluster analyses allow for an in-depth look at parental experiences, these methods are highly interpretive. Central to developing knowledge translation tools is applying the feedback that is stated by end-users; in this case, the end-users are parents. Our study conducted interviews with parents in order to develop narratives for a knowledge translation tool, an e-book, to help parents cope with and manage their child’s chronic pain. As such, we used qualitative description in the analysis for our data. Qualitative description refers to low-inference interpretation in order to provide a comprehensive summary of an event.^14^ We used this method to garner results that closely represented the opinions and thoughts of parents, rather than methods that would yield highly interpretive results, which have potential to distort parents’ experiences.^14–16^ Using qualitative description enables descriptive validity, which is an accurate account of events that others observing the same event would be in agreement with.

To date, there are limited studies that use qualitative descriptive approaches with parents of children with pediatric chronic pain. However, one notable study was conducted by Gaughan et al., who used a qualitative descriptive design to understand parents’ perspectives in caring for a child with a specific type of chronic pain; that is, neuropathic pain.^17^ They found that parents’ experiences were multifaceted, which resulted in negative emotional and lifestyle consequences.^17^ As discussed above, chronic pain in children can have different origins and presentations and, as a result, we felt that it was vital to more broadly understand the perspectives of parents with children who have chronic pain.

The purpose of this study was to describe parents’ experiences with pediatric chronic pain in order to develop a narrative for our knowledge translation tool, an e-book.

## Design

**Figure 1.**
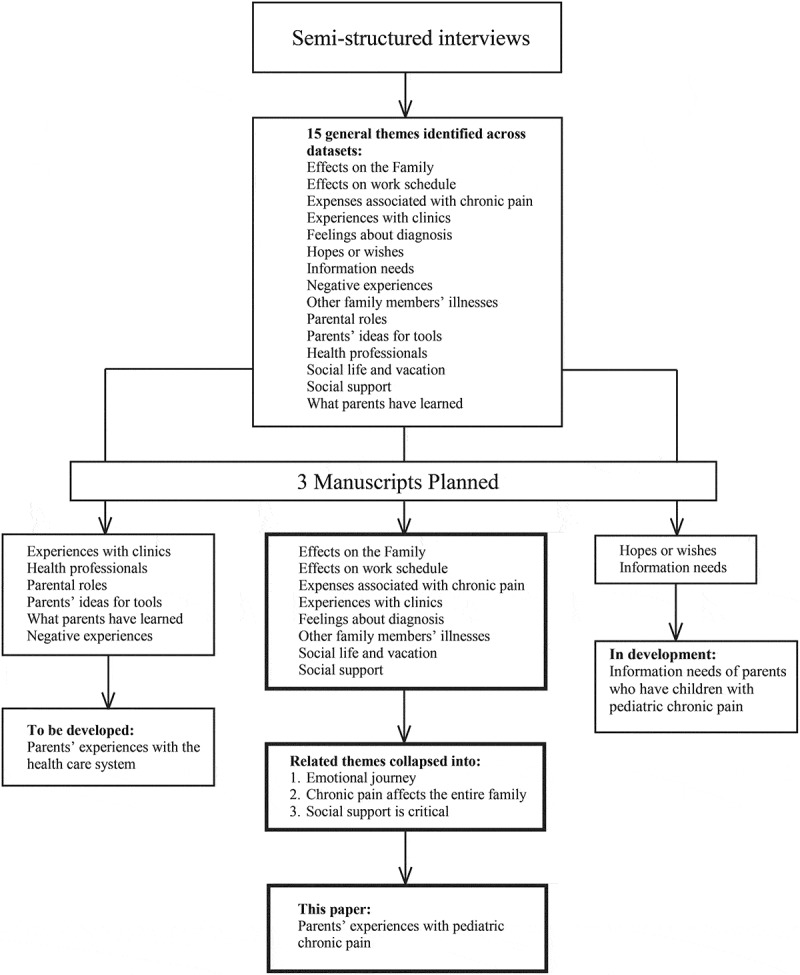
Qualitative analyses.

**Figure 2.**
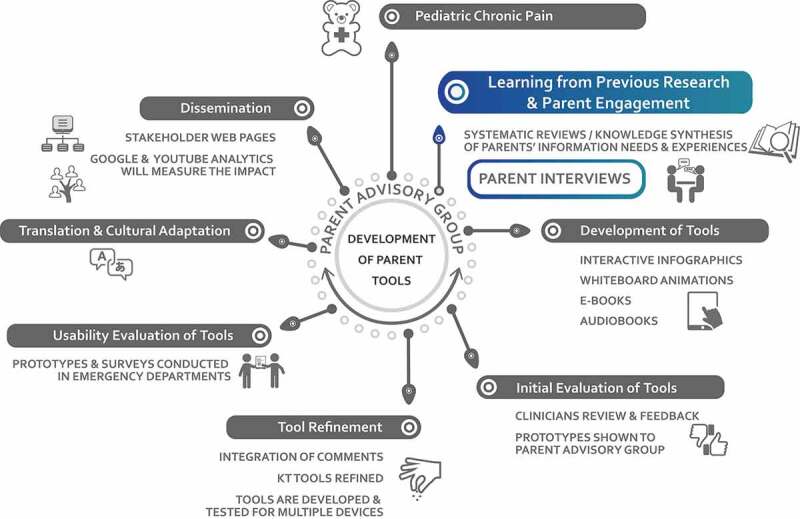
Chronic pain e-book development wheel.

This research study is part of a larger project in which the aims were to work with parents to develop an e-book for families with children with pediatric chronic pain.^18^ Central to developing knowledge translation tools is applying the feedback that is stated by end-users; in this case, the end-users are parents. We conducted interviews with parents to develop narratives for a knowledge translation tool (e-book), to help parents cope with and manage their child’s chronic pain. As such, we employed a qualitative descriptive approach. Qualitative description refers to low-inference interpretation in order to provide a comprehensive summary of an event.^14^ We used this method to garner results that closely represented the perspectives of parents, rather than methods that would yield highly interpretive results, which have potential to distort parents’ experiences.^14–16^ Using qualitative description enables descriptive validity, which is an accurate account of events that others observing the same event would be in agreement with. Through these interviews, a rich data set of parental experiences emerged. As a result, a number of themes were identified in these data postanalyses. Figure 1 illustrates all of these themes and identifies the themes that will be discussed in this article. This article focuses on parental experiences as they relate to the family household. Separate manuscripts will be drafted to present parents’ information needs and parental experiences with the health care system. This study used a qualitative description design. Figure 2 situates the parental interviews (presented in this article) in the process of developing the e-book for parents with a child with chronic pain (results of the e-book development are reported elsewhere).^18^

## Ethics

Ethics approval was received from our local health ethics review board (Pro00050891). Operational approval was received through Alberta Health Services to conduct this study at the Stollery Children’s Hospital Pediatric Chronic Pain Clinic.

## Recruitment

Participants were recruited from the Stollery Children’s Hospital Chronic Pain Clinic in Edmonton, Canada. Inclusion criteria required that participants (1) speak English and (2) have a child under the age of 18 who has chronic pain. Recruitment strategies included posters displayed in the waiting room and distributing information letters from clinical staff to patients and their parents. Eligible parents who were interested in participating in our study first signed a consent form allowing a research coordinator to contact them at a later date. Their information was then forwarded to the research coordinator, who contacted parents within a week with more information regarding the study and interviews were scheduled. Consent forms were signed prior to the start of each interview. Recruitment occurred over 11 months.

## Data collection

Thirteen parents (12 mothers, one father) were recruited and interviewed by a research coordinator trained in qualitative methods. Data were collected through semistructured interviews. Prior to interviews, demographic information was collected from all parents. Table 1 provides participants’ demographic information and information regarding their children’s pain. Interviews, transcription, and analysis occurred concurrently to monitor the progress of interviews and permit follow-up of ideas that emerged from the data. Interviews ranged from 47 to 134 min in length and were recorded on a password-protected device and uploaded to encrypted servers for transcription by a local company. Data were then stored on secure computers for analysis. As categories emerged during data analysis, interviews became more precise and purposeful. Data collection continued until analytic redundancy of major categories was achieved. Data saturation occurred following the 13th interview and, as such, data collection was ended.

**Table 1.** Demographics of parents and caregivers.

	Category	Count (%), *n* = 13
Sex	Male	1 (7.7)
	Female	12 (92.3)
Age group (years)	20–30	0 (0)
	31–40	1 (7.7)
	41–50	9 (69.2)
	51+	3 (23.1)
Marital status	Single/widowed/divorced	2 (15.4)
	Married/common law	11 (84.6)
Annual household income (Canadian dollars)	25 000–49 999	1 (7.7)
	50 000–74 999	1 (7.7)
	75 000–99 999	2 (15.4)
	100 000–150 000	1 (7.7)
	150 000+	3 (23.1)
	I don’t want to share this	4 (30.7)
	I don’t know	1 (7.7)
Highest education level	Some high school	1 (7.7)
	Postsecondary diploma	3 (23.1)
	Postsecondary degree	6 (36.2)
	Graduate degree	3 (23.1)
Relationship to child with pain	Parent	13 (100)
	Other	0 (0)
Age of child who has pain (years)	12 and under	1 (7.7)
	13–17 years	12 (92.3)
On average, how often does the child have pain per week (days)	Every day	11 (84.6)
	5 to 6 days per week	1 (7.7)
	2 to 4 day per week	1 (7.7)
	1 day per week	0 (0)
	Less than 1 day per week	0 (0)
How much school does the child miss each week due to pain (days)	Rarely miss	3 (23.1)
	½ day	1 (7.7)
	1 day	4 (30.7)
	2 days	2 (15.4)
	3 days	1 (7.7)
	4 days	0 (0)
	5 days	1 (7.7)
	I don’t know	1 (7.7)
Single or multiple source of pain	Single	6 (46.2)
	Multiple	7 (53.8)
Child’s source(s) of pain	Abdominal	1 (7.7)
	Muscular	2 (15.4)
	Pelvic	1 (7.7)
	Nerve	1 (7.7)
	Arthritis	1 (7.7)
	Headache, abdominal	5 (38.5)
	Headache, abdominal, muscular	1 (7.7)
	Headache, muscular	1 (7.7)

Following each interview, the interviewer wrote in-depth field notes about each interview, making notes of details regarding the setting, participant, observational content, theoretical content, methodological content, and reflexive content. This was done in order to assess the progress of the interviews, assess which strategies were working well and which ones were not, and keep track of emerging themes and insights.

The following questions guided the interview:

1. Tell me about your experience of having a child with chronic pain.
2. Tell me about your child’s experience with having chronic pain (e.g., Did it affect his or her social activities? Physical activities? Learning ability at school?)
3. How old was your child when he or she was diagnosed with chronic pain?
4. How did you feel during this experience? (probe at the events)
5. With these experiences that you and your child have had with chronic pain, what would you teach health care professionals about your needs as a parent? Your child’s needs?
6. What strategies were put in place by health care professionals to help you or your child? (For example, did they use medication? Provide you with education about chronic pain? Other strategies?)
7. Before the chronic pain diagnosis, how did you manage your child’s pain? How are you managing your child’s chronic pain now? (Any techniques that you use; for example, distraction, talking with your child, holding your children, etc.)
8. Are there any other comments or stories that you would like to share?

## Data analysis

Data were managed using the NVivo 11 (NVivo qualitative data analysis Software: QSR International Pty Ltd. Version 11, 2015) and data analysis occurred in three phases: coding, categorizing, and developing themes.

### Coding

First, all data were coded to facilitate the analysis. The code words reflected the essence of the data. Codes were operationally defined so that they could be used throughout. Coding was done jointly by the principal investigator and projector coordinator, who coded the data separately and met to ensure that there was consensus between both data sets.

### Sorting and categorizing

Codes were then placed into broad categories (major unit of analysis). As categories emerged, their theoretical properties were defined. Two or more categories were compared in an effort to locate similarities and differences between them.

### Themes

When categories were synthesized into themes, a broad, comprehensive, and holistic view of the data was obtained. NVivo 11’s search and retrieval capacity facilitated category development as well as coding, retrieval, and comparison of coded data.

Four criteria guided the credibility or trustworthiness of the research process: (1) credibility, (2) confirmability, (3) dependability, and (4) transferability.^19^ First, we addressed credibility by broadly sampling interview participants to ensure that we did not have an overconcentration of participants with particular attributes or experiences. Second, we addressed confirmability of our findings through keeping a comprehensive audit trail that documented all conclusions, interpretations, and recommendations arising from the data. In addition, detailed accounts of all of the “raw” data, specifically field note jottings, individualized interview schedules, and interview tapes, were kept. A complete inventory of all analysis products, which included written-up detailed field notes and theoretical and analytical memos that document developing thoughts about the data, was logged. Third, dependability or consistency was addressed by keeping a detailed audit trail that documents all of the decisions that were made throughout the research process, selection of the qualitative descriptive approach, and involvement of more than one coder. Finally, transferability was addressed by providing thick description of the parents’ experiences. Thick description is a process in which a qualitative researcher provides detailed accounts of his or her experiences during data collection, including information about behaviors as well as context. This is important because transferability depends on the similarities between the sending and receiving contexts. Thus, detailed descriptions of the parents’ experiences were collected and reported with sufficient detail and precision to allow the reader to make judgments about transferability.^20^

## Results

Publication convention (manuscript length) prevents the publication of all qualitative analyses in one manuscript due to the rich detail of the data. As a result, we had to strategically plan the publication of these analyses. Figure 1 depicts how the initial 15 general themes are being presented in three manuscripts.

Eight general themes were initially created that focused on the parental experiences with pediatric chronic pain. Further analysis of these data resulted in the collapsing of related themes into three final themes: parents’ struggles to cope with and navigate the system (“emotional journey”), chronic pain affects the entire family, and social support is critical (Figure 1). These themes are reported here. The other themes not related to parental experiences will be reported elsewhere in manuscripts in development.

## Parents’ emotional journeys

Parents described a variety of different emotions that highlighted the difficulties and struggles they had in coping with their child’s condition along with the difficulties in navigating the system to help their child get better. These emotions included frustration, anger, desperation, helplessness, sadness, worry, feeling overwhelmed, and guilt. These negative emotions were due to many different factors from early in their child’s illness trajectory. Parents expressed worry and concern because they sought treatment for their child’s pain yet observed minimal improvements. As the pain continued over time and treatments and interventions still provided little progress, parents described feeling hopeless. Many expressed that the relentless cycle of referrals from health care provider to health care provider caused feelings of desperation in finding an effective treatment. Some parents described how alone they felt during this process:

… There’s a feeling of helplessness. … When a kid has chronic pain, it’s just not solved by a Tylenol or something like that. … It’s overwhelming. You feel helpless, like there isn’t really any help anywhere. Is this kinda how your kid has to live? (Parent 6)

For many families, finding a diagnosis took many years, during which parents watched their children experience pain. Parents stated that they often felt guilty and sad because they could not provide the tools to their child to help them feel better. As such, this has potential to impact parents’ psychological health.

And I feel guilty, with a tremendous amount of guilt. It’s my job to give her all the tools she needs, to be her own—whatever her own is. And I don’t feel like—since chronic pain has … affected her life—that I’ve got the tools. I don’t even know where to look for them. I mean, some of them have fallen in my lap, is all that’s happened. But I still don’t have one person saying, “Okay, yeah, and now the next step would be to do this.” “Oh, okay.” You know? […] So I still feel very … lost. (Parent 15)

Most parents described feeling frustrated. Reasons for their frustration included feeling that they were not being heard, feeling that their children were unable to partake in certain activities, not knowing what was wrong with their child, not knowing how to get their child help, and frustrations with the medical system and the wait times.

It’s … frustration with the disease. Frustration with the medical system sometimes … with myself. … I think it’s just the frustration with the situation sometimes … It’s a chronic issue. It’s never gonna go away. (Parent 2)

Once their children were diagnosed and treatment was started, parents further worried about their children’s future care. Specifically, they worried about what would happen once their children were no longer eligible to be treated at the Pediatric Chronic Pain Clinic. They were concerned with how their child would transition into adulthood, about how they would be able to cover the costs of treatments, and about how they would be treated in the health system as an adult. One parent stated:

Now that she’s turned 17, it’s a little bit scary. I am more worried, yes I am. The adult world is a whole [lot] different and they don’t understand that the time when she really needs to get her care is now—not wait in twelve hours, because it’s past the point that it’s gonna help her, yet I understand a person having a heart attack is very important, and they need to be looked after. But it’s—and they won’t see that. They’ll have a tough time. (Parent 4)

Notably, despite the concerns about future transitions into adult care, some parents expressed relief in finally obtaining a chronic pain diagnosis. The relief allowed parents to finally focus on helping to improve their child’s condition, rather than continuously searching for answers.

I would say she felt relieved—’cause it’s validation that it’s not all in her head. And I think that has to do with the social pressure that there was. (Parent 15)

When all those doctors are in there, and everybody’s listening to her and saying, “[…] We believe you. It’s not in your head, we believe you.” That was a relief. (Parent 7)

## Chronic pain affects the entire family

We developed three subthemes in our analysis of how chronic pain impacts a family. These subthemes were (1) impact on social life, including vacation, (2) impact on employment, and (3) impact on family finances.

### Social life and vacation

Parents described having a child with chronic pain as being difficult for their family.

Chronic pain is an unpredictable condition because symptoms can be irregular and often occur unexpectedly. As a result, family activities were dictated by the child’s condition on any particular day. Parents were expected to be flexible in their planning of social activities, which is often difficult for busy families with multiple children and responsibilities. One parent described:

We’ve had tickets to so many concerts, that we never made it to. You know, we buy these tickets. And my husband and I are planning we’re gonna go together. And he [son] ends up sick, and my husband takes my other son, or my husband goes with a friend and I stay home with [child], so … it does impact in that way. (Parent 2)

Because of the uncertainty and sporadic nature of the illness, families described being hesitant to make commitments. In several cases, other individuals outside the family may be impacted by the child’s chronic pain such that they too must be prepared for parents of children with pediatric chronic pain to cancel or modify plans.

So, just even things like we’re supposed to go on a trip, or we hummed and hawed for a long time […] to even say yes. But you say yes, with the caveat, knowing that things may change. (Parent 3)

Parents told accounts of being too exhausted and jealous of other families, resulting in isolation of isolation of their families from social networks. One parent expressed feeling too exhausted to partake in certain activities:

We’re exhausted. […] We’ve shut down who we deal with. And I’m sure it happened gradually, but I’m sure it wasn’t that easy on her either. Like all of a sudden you wake up one day and “I’ve completely isolated my family.” (Parent 8)

The parent explained how it is difficult to socialize with healthy individuals because of jealousy over the fact that their child could no longer participate in activities they were once able to participate in.

You don’t wanna deal with people that are healthy. There’s no way we’re gonna go out there and not feel jealous, right? We used to live at the lake and she played basketball, she played soccer. She was in swimming, like—we got to go, go, go. Now you go out, everybody gets to go except you, right? … You get sick of seeing healthy people when you always feel like a pack of dirt. (Parent 8)

### Vacations

Some parents found it difficult to make vacation plans because of the amount of planning and accommodations that must be taken into consideration for the child with chronic pain. For example, one family was required to schedule frequent stops on their road trip in order to allow their child to rest and another parent was required to spend an entire trip abroad in a hotel with her child due to the pain. Parents were also deterred from traveling with their child with chronic pain because of the additional costs that were potentially required (e.g., transporting oxygen or making more comfortable travel arrangements for their child).

For example, this summer, we had to go down to southern [province], ah, for family wedding. While going down there, we planned on taking two days and it did take us the full two days, because we did it in pieces and stay overnight and halfway and … [wife] had to work on the Monday. So we drove all the way Sunday. And so we’re looking at that and we knew, no way, you know, [daughter] could make that trip—Coming back the whole way. So we left her down there. And with family. And then they put her on the airplane a few days later and she just flew back, so … I guess that’s an added expense that we wouldn’t have. (Parent 12)

### Expenses

Many spoke about the expenses associated with having a child with chronic pain. Medications and therapies (such as physiotherapy and acupuncture) not covered by the health care system can add up. Furthermore, for families who live in rural areas and must travel to the city for appointments and care, gas and parking costs can become a burden, particularly for families who have several appointments in a given week.

Physio appointments were 95 dollars a time—not cheap […] and I wasn’t sure how we were gonna make it work. (Parent 13)

Um, it’s hit us in the pocketbook pretty good because we’ve had to have chiropractor and massage, ’cause we thought it would help it. It seemed to be helping, but it was very short-term help. […] We were paying out of pocket. Now the trips to the city and stuff. […] It’s a lot. (Parent 7)

Travel accommodations often had to be made for the child with chronic pain. One family described a family trip in which all other members of the household traveled by car but the child with chronic pain required travel via airplane. Another family described the expenses associated with paying for oxygen on a flight.

So—and flying is extremely difficult—very expensive. It’s just killer, what you have to pay for oxygen on the flight. (Parent 6)

### Employment

Another financial sacrifice for families with a child with chronic pain was related to employment. Many parents explained that they could not work as much given the unpredictable nature of the illness and the constant appointments. Many parents discussed having to be “on call” in the event that their child’s pain flared up. Parents who had flexible jobs agreed that the unpredictable nature of chronic pain was easier for them to manage:

I work casually. And I would have been able to work more, but what I do is I stay home, because if in the morning if he’s bad, then he might have to miss the morning, but then he can go in the afternoon. Or like every hour I’m saying, “okay, science is next. Do you think you can go to science? No? Okay, let’s—let’s try for the next period, right, of school.” […] I have to work a certain number of shifts in six months. And I think I just made it. For these past six months. But that’s all I can do. Um, yeah. Because I—he—we tried to make him go to school as much as he can, but it means me being home. (Parent 9)

I’m very fortunate my job is quite flexible, but I often get phone calls, I often get called to the school. It’s that living—just hoping that you’re gonna get through one day without a phone call or an issue. So I find it … it can be really exhausting. (Parent 2)

Many parents described having to make career sacrifices to care for their child with chronic pain. One mother stated that she had to stop going to work conferences, particular those far away. Another mom described working casual hours, and others had to quit their jobs completely. One parent described having to put his education and job search on hold in order to provide care for his child.

## Social support was critical

Social support from family, friends, health professionals, teachers, and coworkers was critical for these parents. Many parents discussed family as an important source of social support. One family’s grandmother is also afflicted with chronic pain; as a result, she taught the child certain strategies and techniques to alleviate the pain. Other parents talked about how their families would conduct research on how to manage the condition and offer advice and feedback. Parents discussed other siblings in the household taking on more responsibility to assist the child with chronic pain or checking up on the child when mom and dad are busy.

My husband’s family lives in [country]. But my—my parents and family are here, and they’re very, very supportive. […] And then a friend of mine, his daughter also has gone through this. And so, kind of hearing, you know, other people’s stories has—has helped. (Parent 9)

So at that time, we were fortunate that my oldest daughter already drove. So she didn’t have to take the bus to school. And that made a huge difference. So all the headache problems, with noise and all the rest, decreased, because [daughter] wouldn’t have that headache that was three times worse because of the noise and the rambunctiousness of the bus. So that was fortunate, that she was able to have that person to drive. (Parent 4)

For some families with relatives who live far away, emotional support was reported to help families.

I don’t feel that we have enough support here. […] But I have like a lot of emotional support from my family. Just not a lot of concrete—“I’ll help you take the children to an appointment” support. (Parent 2)

Likewise, parents expressed that friends are important when caring for a child with chronic pain. One parent described her friends as helping her find the Chronic Pain Clinic and motivating her child to be an active participant in her own care. Others describe having friends who provide suggestions and “get it.”

For example, the one friend’s the one that got me here [referring to the Chronic Pain Clinic]. Another friend got me to the naturopath [alternative medicine]. Another friend’s good for kicking [daughter] on the bum. […] I used to have a friend who’s has some sort of joint problem. And she knows what it is. […] So she can relate to [daughter.] Like they actually have conversations about their pain, and she knows—she’s taking a Motrin [ibuprofen and naproxen] or [daughter’s] taking this. And if [daughter’s] not feeling well, she’s like, “Well, do you need something? Because I can give you a Motrin, because that’s what I take.” … People have been really supportive. (Parent 17)

She’s [friend] on—speed dial. And she’s really good. Like she’ll just sort of balance me out. […] [She] is a good one for me to bounce things off of—just because of the experience. ’Cause not everybody gets it. They don’t. They just don’t get it. And in some ways initially, you expect them to get it—when you realize that they don’t have that experience. They just don’t get it, so it’s good to have those to bounce off. It just makes a difference. (Parent 3)

## Discussion

This study adds to the body of literature suggesting that in families of children with chronic pain, there are marked effects on the rest of the family, including parents. Several key factors were identified that are relevant to the health and well-being of youth with chronic pain and their families., We developed three key themes from parental interviews: having a child with chronic pain affects the whole family, parents struggle to cope and navigate the system when their child has a chronic pain condition, and social support is critical to these families. These themes are relevant to young people with chronic pain and their parents and families and have implications for health- and quality of life–related outcomes in the young people with pain, their parents, and those around them. Importantly, these findings fit quite well within established research evidence and have potentially important implications for future research and translation into treatment and materials for families of youth with chronic pain.^21,22^

Parents in our study identified several important effects of their child’s chronic pain on themselves and their family life. These effects included broad impacts on health and quality of life as well as disrupted goals and commitments. It also affected families’ abilities to engage in valued activities and healthy coping activities that could potentially mitigate the negative effects of chronic pain. Health effects included exhaustion, poorer mental health, increased stress, and a markedly increased workload associated with caring for a very unwell child. This burden included engagement in multiple health care appointments for diagnosis and treatment while attempting to meet the demands of many other personal, family, and work-related commitments. Specific effects include parents’ struggles with completing employment-related tasks and requirements. Some parents also highlighted the negative impact of this factor on family finances. In addition, some parents emphasized the effects on families’ abilities to engage in recreational activities, including preferred activities and vacations. There is increasing evidence that such activities promote better physical and mental health.^23^ Compounding the deficit of time for adequate self-care is the tremendous burden that parents experience as a result of caring for a child experiencing a high level of pain-related disability. A related finding was reported by Gaughan et al. in their study of nine parents whose children had chronic neuropathic pain.^17^ The parents identified feeling burdened trying to get through the day when unable to resolve their child’s pain. In addition, parents identified how the child’s pain controlled the whole family. Our findings also complement and extend findings from a study by Jordan et al. in which parents described living a life in limbo, in which their lives were often “on hold” due to the demands associated with their children’s health conditions, at the expense of their own happiness.^7^

The parents in our study identified many negative emotions, including frustration, anger, and fear. These findings also complement and extend Jordan et al.’s findings related to the importance of parental feelings of helplessness, containment, balance, and re-evaluation.^8^ These emotions were also similar to those reported in the study by Maciver et al.^10^ In that study, 12 parents whose children had various chronic pain conditions, including musculoskeletal pain and neuropathic pain, were interviewed. The parents identified feeling frustrated by their perceived failure to help their children manage distress. They also highlighted that this led to the loss of their expected parenting role. The effects of youth chronic pain on their families and the associated experience of such a broad range of negative emotions in parents, including the intensity of those emotions, must not be taken lightly. There is ample and steadily growing evidence that the long-term experience of such severe emotional states can have devastating effects on the health and quality of life of these parents.^24,25^ Further, other work highlights the potential negative effects of parental emotional turmoil and worse mental health on their children, including children’s physical and emotional health.^26,27^ Particularly salient negative emotions worth noting in parents included severe anxiety arising following extensive medical consultations and treatments that resulted in neither diagnostic clarification nor health improvement for their children. In addition, significant hopelessness, associated with the perception that no help was available for their children, was highlighted by parents. Parents also expressed intensified feelings of hopelessness and anxiety associated with their perception of their children’s poor long-term prognosis due to the negative impact of unresolved chronic pain. In contrast to the hopelessness expressed was parents’ relief that occurred following later diagnosis, treatment plan formulation, and learning about best practice methods for managing their children’s pain.

Of considerable further importance was the theme noted that parents found social support from a number of sources to be critical for a number of reasons. Key sources of support included immediate and extended family, friends, health care providers, and work colleagues. Benefits arising from this social support included the sharing of coping strategies, including the teaching of active self-management strategies, shared educational resources, and social modeling of healthy coping. Parents also reported reduced individual burden associated with the efforts of others to assist with the completion of a variety of tasks in the form of sharing the load. In contrast, parents reported increased emotional pain arising from social isolation as well as the invalidation or rejection of their child’s condition. Unfortunately, social isolation was a common experience of parents in this study, arising from a perceived lack of time to engage socially as well as physical and mental exhaustion associated with the considerable burden experienced by these parents. Social support for parents is critical to their health and potentially critical to the health of their families, including their children with chronic pain.^1^

In addition, parents in our study identified the negative effects associated with the lengthy time it took to receive a diagnosis. For some families, this took several years. It appears that the considerable time to diagnosis may have resulted from a variety of factors. This includes some health care providers’ biases toward carrying out multiple diagnostic investigations as part of a search for a cure for pain, even after appropriate medical investigations had been completed. In addition, there continues to be a lack of awareness of best practice standards for managing chronic pain among primary care and specialist health care providers. In our study, parents described feeling guilty and sad that their child suffered in pain while awaiting a diagnosis and that they struggled to help their child because they did not have the tools to help their child manage pain. A previous study by Reid et al. of 14 parents who attended a pediatric chronic pain clinic found that 93% identified the need for information about the cause of their child’s pain and also noted that treatment options were important to them.^28^

Parents in the present study also reported anxiety about how their child would transition into adulthood, including their child’s ability to obtain appropriate medical care in the adult health system. Given the literature reporting that chronic pain in adolescence is a predictor for ongoing chronic pain in adulthood, this is a very important area to address.^29^ A recent review by Forgeron et al. identified unique factors related to this transition when living with chronic pain, including stigma and disbelief, the individuality of chronic pain, and the lack of formal education of non-pain clinicians.^30^

There are some noteworthy limitations associated with the current study. Given that parents were reflecting on previous events, recall bias may be evident. It may also be that participants who volunteered for this study were particularly motivated to share their experiences due to the severity of the difficulties that they faced, whereas those who opted not to participate in our study may have had different experiences. Finally, we were only able to interview one father in this study and, as such, views expressed in this study may not be indicative of fathers’ experiences with pediatric chronic pain.

Notwithstanding these limitations, this study also includes several important strengths. These include the use of careful and rigorous methodology using high standards of data collection and analysis to optimize credibility, confirmability, dependability, and transferability. Though the demographics of the sample reported here are in line with a typical North American middle-class population, the findings are in line with those of other similar studies carried out internationally.^6,7^ In addition, the findings of this study highlight the importance of the adoption of best practice standards for chronic pain management among primary care and specialist health care providers. Once high-quality and appropriate medical diagnostic processes and investigations have been completed, a move toward a diagnosis of chronic pain, education related to the neurobiological underpinnings of the disorder, and cessation of the exhausting search for medical answers is important. Unfortunately, neurobiological phenomena underlying the presentations of complex chronic pain cannot presently be adequately evaluated by current medical technology. A turn toward a holistic biopsychosocial model of chronic pain self-management is further supported by parental reports in this study.

Arguably, the most important outcome of this study is the potent reminder of the significant effects of chronic pain on a young person and that person’s parents and family. Given the high prevalence of pain in children and adolescents, this further highlights the widespread impact of chronic pain in children on society. This effect is almost certainly amplified over time because the trajectory of the child’s development, ability to engage in academic training, and later enter the workforce is at considerable risk of being affected by the negative consequences of pain. Further, though society’s perception of human development is inherently biased toward the early years of life, broadly thinking researchers including Erikson noted key developmental tasks that occur across the life span to the end of life.^31^ It is entirely possible that another significant loss to individuals and society results from the long-term effects of chronic pain in young people on their long-term physical and mental health as well as the productivity of parents and family members. Our findings echo previous calls to direct additional attention and resources toward translational family-centered programs for this large group of highly at-risk individuals. Given the bidirectional effects exerted by chronic pain and the chronic stress associated with it on these youth and their parents, ongoing efforts toward further developing and refining multidisciplinary programs promoting best practice treatments, education, and psychologically based treatment protocols emphasizing awareness- and acceptance-based treatments are warranted. Such programs have the potential to respond effectively. Jordan et al. highlighted themes of the importance of fostering actions aimed at enhancing control and coherence in the lives of these young people and their families.^7^

This article explores the home-life experiences of parents in caring for a child with pediatric chronic pain. Further research should be conducted to explore parents’ experiences within the health care system and parents’ information needs in that context and in general. Furthermore, it may be valuable to interview children with chronic pain to gain their perspectives on how their condition affects their families.

## Conclusion

Though much remains to be learned and studied, this study highlights the importance of the effects of chronic pain in children on their parents and families. It also points to the potential importance of family supports. Another background issue is highlighted related to the ongoing need of education for primary care and specialty health care providers regarding best practice standards associated with the diagnosis and treatment of chronic pain. Of critical importance is the potential effect of these difficulties on the trajectories of the development of a child with chronic pain, his or her parents, and the parent–child relationship longitudinally.^1,21,22^ The effects of these changes on this group of individuals and society cannot be underestimated. Translating these findings and using them to provide more effective interventions in primary and speciality care including in pediatric chronic pain programs is of tremendous potential importance.

## References

[1] Palermo TM, Valrie CR, Karlson CW. Family and parent influences on pediatric chronic pain a developmental perspective. Am Psychologist. 2014;69(2):142–52. doi:10.1037/a0035216. PMC4056332 24547800

[2] Merlijn V, Hunfeld JAM, van der Wouden JC, Hazebroek-Kampschreur A, Passchier J, Koes BW. Factors related to the quality of life in adoolescents with chronic pain. Clin J Pain. 2006;22(3):306–15. doi:10.1097/01.ajp.0000177509.93523.68. 16514332

[3] Roth-Isigkeit A, Thyen U, Stö Ven H, Schwarzenberger J, Schmucker P. Pain among children and adolescents: restrictions in daily living and triggering factors. Pediatrics. 2005;115(2):e152–e162. doi:10.1542/peds.2004-0682. 15687423

[4] Eccleston C, Bruce E, Carter B. Chronic pain in children and adolescents. Paediatr Nurs. 2006;18:30–33. 10.7748/paed.18.10.30.s21 17193918

[5] Kashikar-Zuck S, Powers SW, Vaught MH, Hershey AD, Goldschneider KR. Depression and functional disability in chronic pediatric pain. Clin J Pain. 2001;17:341–49. 11783815 10.1097/00002508-200112000-00009

[6] Noel M, Beals-Erickson SE, Law EF, Alberts NM, Palermo TM. Characterizing the pain narratives of parents of youth with chronic pain. Clin J Pain. 2016;32(10):849–58. doi:10.1097/AJP.0000000000000346. 26736026 PMC4935638

[7] Jordan AL, Eccleston C, Osborn M. Being a parent of the adolescent with complex chronic pain: an interpretative phenomenological analysis. Eur J Pain. 2007;11:49–56. doi:10.1016/j.ejpain.2005.12.012. 16458550

[8] Jordan A, Crabtree A, Eccleston C. ‘You have to be a jack of all trades’: fathers parenting their adolescent with chronic pain. J Health Psychol. 2016;21(11):2466–76. doi:10.1177/1359105315580461. 25897044

[9] Jordan A, Eccleston C, McCracken LM, Connell H, Clinch J. Research papers: the Bath Adolescent Pain–Parental Impact Questionnaire (BAP-PIQ): development and preliminary psychometric evaluation of an instrument to assess the impact of parenting an adolescent with chronic pain. Pain. 2008;137:478–87. doi:10.1016/j.pain.2007.10.007. 18035497

[10] Maciver D, Jones D, Nicol M. Parents’ experiences of caring for a child with chronic pain. Qual Health Res. 2010;20(9):1272–82. doi:10.1177/1049732310367499. 20406993

[11] Eccleston C, Crombez G, Scotford A, Clinch J, Connell H. Adolescent chronic pain: patterns and predictors of emotional distress in adolescents with chronic pain and their parents. Pain. 2004;108(3):221–29. doi:10.1016/j.pain.2003.11.008. 15030941

[12] Hunfeld JAM, Perquin CW, Duivenvoorden HJ, et al. Chronic pain and its impact on quality of life in adolescents and their families. J Pediatr Psychol. 2001;26(3):145. doi:10.1093/jpepsy/26.3.145. 11259516

[13] Smith JA. Beyond the divide between cognition and discourse: using interpretative phenomenological analysis in health psychology. Psychol Health. 1996;11(2):261–71. doi:10.1080/08870449608400256.

[14] Sandelowski M. Whatever happened to qualitative description? Res Nurs Health. 2000;23:334–40. 10940958 10.1002/1098-240x(200008)23:4<334::aid-nur9>3.0.co;2-g

[15] Sandelowski M. Whatʼs in a name? qualitative description revisited. Res Nurs Health. 2010;33(1):77. 20014004 10.1002/nur.20362

[16] Neergaard MA, Olesen F, Andersen RS, Sondergaard J. Qualitative description—the poor cousin of health research? BMC Med Res Methodol. 2009;9(1):52. doi:10.1186/1471-2288-9-52. 19607668 PMC2717117

[17] Gaughan V, Logan D, Sethna N, Mott S. Parents’ perspective of their journey caring for a child with chronic neuropathic pain. Pain Manag Nurs. 2014;15(1):246–57. doi:10.1016/j.pmn.2012.09.002. 23219393

[18] Reid K, Hartling L, Ali S, Le A, Norris A, Scott SD. Development and usability evaluation of an art and narrative-based knowledge translation tool for parents with a child with pediatric chronic pain: multi-method study. J Med Internet Res. 2017;19(12):e412. doi:10.2196/jmir.8877. 29242180 PMC5746621

[19] Korstjens I, Moser A. Series: practical guidance to qualitative research. Part 4: trustworthiness and publishing. Eur J Gen Pract. 2018;24(1):120–24. doi:10.1080/13814788.2017.1375092. 29202616 PMC8816392

[20] Firestone WA. Alternative arguments for generalizing from data as applied to qualitative research. Educ Res. 1993;22(4):16–23. doi:10.3102/0013189X022004016.

[21] Craig KD. Social communication model of pain. Pain. 2015;156(7):1198–99. doi:10.1097/j.pain.0000000000000185. 26086113

[22] Simons LE, Smith A, Kaczynski K, Basch M. Living in fear of your child’s pain: the parent fear of pain questionnaire. Pain. 2015;156(4):694–702. doi:10.1097/j.pain.0000000000000100. 25630026 PMC4366345

[23] Coleman D, Iso-Ahola SE. Leisure and health: the role of social support and self-determination. J Leisure Res. 1993;25(2):111–28. doi:10.1080/00222216.1993.11969913.

[24] DeLongis A, Folkman S, Lazarus RS. The impact of daily stress on health and mood: psychological and social resources as mediators. J Pers Soc Psychol. 1988;54:486–95. 3361420 10.1037//0022-3514.54.3.486

[25] Bottaccioli AG, Bottaccioli F, Minelli A. Stress and the psyche-brain-immune network in psychiatric diseases based on psychoneuroendocrineimmunology: a concise review. Ann N Y Acad Sci. 2018. doi:10.1111/nyas.13728. 29762862

[26] Cousino MK, Hazen RA. Parenting stress among caregivers of children with chronic illness: a systematic review. J Pediatr Psychol. 2013;38(8):809–28. doi:10.1093/jpepsy/jst049. 23843630

[27] Golfenshtein N, Srulovici E, Medoff-Cooper B. Investigating parenting stress across pediatric health conditions—a systematic review. Issues Compr Pediatr Nurs. 2015;1–49. doi:10.3109/01460862.2015.1078423. 26367769

[28] Reid K, Lander J, Scott S, Dick B. What do the parents of children who have chronic pain expect from their first visit to a pediatric chronic pain clinic? Pain Res Manage. 2010;15:158–62. 10.1155/2010/958792 PMC2912615 20577658

[29] Hassett AL, Hilliard PE, Goesling J, Clauw DJ, Harte SE, Brummett CM. Reports of chronic pain in childhood and adolescence among patients at a tertiary care pain clinic. J Pain. 2013;14(11):1390–97. doi:10.1016/j.jpain.2013.06.010. 24021576

[30] Forgeron P, Higginson A, Truskoski C. Departure from pediatric care: transitioning of adolescents with chronic pain to adult care. Pain Manag Nurs. 2017;18(5):273–77. doi:10.1016/j.pmn.2017.05.001. 28778412

[31] Erikson EH, Erikson JM. The life cycle completed (extended version). New York (NY): W. W. Norton; 1998.

